# Amyloid Beta Annular Protofibrils in Cell Processes and Synapses Accumulate with Aging and Alzheimer-Associated Genetic Modification

**DOI:** 10.4061/2009/689285

**Published:** 2009-07-14

**Authors:** Hideko Kokubo, Rakez Kayed, Charles G. Glabe, Matthias Staufenbiel, Takaomi C. Saido, Nobuhisa Iwata, Haruyasu Yamaguchi

**Affiliations:** ^1^School of Health Sciences, Gunma University, 3-39-15 Showa-machi, Maebashi, Gunma 371-8514, Japan; ^2^Geriatrics Research Institute and Hospital, 3-26-8 Ootomo-machi, Maebashi, Gunma 371-0847, Japan; ^3^Department of Neurology, University of Texas, Medical Branch, Galveston, TX 77555, USA; ^4^Department of Molecular Biology and Biochemistry, University of California, Irvine, Irvine, CA 92697-3900, USA; ^5^Nervous System Department, Novartis Institutes of Biomedical Research, CH-4002 Basel, Switzerland; ^6^Laboratory for Proteolytic Neuroscience, RIKEN Brain Science Institute, 2-1 Hirosawa, Wako-shi, Saitama 351-0198, Japan

## Abstract

Amyloid *β* (A*β*) annular protofibrils (APFs) have been described where the structure is related to that of *β* barrel pore-forming bacterial toxins and exhibits cellular toxicity. To investigate the relationship of A*β* APFs to disease and their ultrastructural localization in brain tissue, we conducted a pre-embedding immunoelectron microscopic study using anti-annular protofibril antiserum. We examined brain tissues of young- and old-aged amyloid precursor protein transgenic mice (APP23), neprilysin knockout APP23 mice, and nontransgenic littermates. *α*APF-immunoreactions tended to be found (1) on plasma membranes and vesicles inside of cell processes, but not on amyloid fibrils, (2) with higher density due to aging, APP transgene, and neprilysin deficiency, and (3) with higher positive rate at synaptic compartments in aged APP23, especially in neprilysin knockout APP23 mice. These findings imply that APFs are distinct from amyloid fibrils, interact with biological membranes, and might be related to synaptic dysfunction in Alzheimer model mouse brains.

## 1. Introduction

The pathologic hallmarks of Alzheimer's disease (AD) are accumulation of senile plaques and neurofibrillaly tangles, and loss of synapses and neurons. Amyloid beta protein (A*β*) is the principal component of senile plaques and accumulates in brain. In the process of accumulation, A*β* aggregates from monomer to oligomers, protofibrils or fibrils. Recently, prefibrillar aggregates of A*β*, such as spherical prefibrillar oligomers (PFOs) [1], A*β*-derived diffusible legands (ADDLs) [2, 3] and protofibrils [4–6], have been a focus of A*β*-derived neurotoxicity [7, 8]. 

Dysfunction of synaptic plasticity and integrity is the typical and early function-related event in AD [9]. Soluble A*β* oligomers have been considered primarily responsible for impaired synaptic plasticity and cognitive dysfunction prior to the formation of senile plaques in transgenic mice overexpressing human-type mutant amyloid precursor protein (human APP Tg mouse) [10–12]. The soluble A*β* concentration in brain shows a stronger correlation with cognitive dysfunction [13, 14] and synapse loss [13, 15]. A*β* PFOs can assemble into annular protofibrils (APFs) [16]. APFs have been described as ring-shaped or pore-like structures. The structure of APFs is related to that of *β* barrel pore-forming bacterial toxins and exhibit cellular toxicity, although the toxicity of APFs is less than that of PFOs [6, 16, 17]. 

 Neprilysin is the major A*β*-degrading enzyme in brain and contributes to clearance of A*β*, especially oligomeric A*β*, in mouse brain [18]. The reduced activity of neprilysin leads to the elevation of A*β* PFOs at the synapses, and the impaired hippocampal synaptic plasticity and cognitive function [12]. Therefore, it is worthwhile to investigate the relation between oligomeric A*β* and neprilysin to AD pathology using human APP transgenic (Tg) mice with or without neprilysin deficiency. 

Previous studies on APFs were mainly conducted using biochemical or biophysical methods, and cell culture systems. However, culture cells do not exactly reflect the physiological condition in vivo, and the ultrastructural localization of APFs in brain tissue has not yet been demonstrated. The purpose of this study is to clarify the ultrastructural localization of APFs in brain tissue and its relation to Alzheimer-associated genetic modification using pre-embedding immunoelectron microscopy (IEM). We used the anti-annular protofibril (*α*APF) antiserum [16] and Alzheimer model Tg mice [12]. Our study first provides ultrastructural evidence that (1) APFs localize to plasma membranes and vesicles inside of cell processes, (2) APFs are on a distinct pathway from amyloid fibril formation, (3) APFs increase with aging, APP transgene, and neprilysin deficiency, and (4) the synaptic compartment in aged APP Tg mice, especially in neprilysin deficient ones, shows higher accumulation of APFs.

## 2. Materials and Methods

### 2.1. Animals

In this study, young (3.5–4 month-old) and aged (17–20 month-old) APP Tg mice with human-type APP carrying double mutations (APP_K670N/M671L_; APP23) (NEP^+/+^APP^+^) [19], homozygous neprilysin (NEP)-deficient APP23 mice (NEP^−/−^APP^+^) [12], and nontransgenic (NEP^+/+^APP^−^) littermates were used. NEP^−/−^APP^+^ and NEP^+/+^APP^+^ mice were produced by breeding NEP^+/−^APP^+^ and NEP^+/−^APP^+^ mice [12]. All mice were on the same genetic background (C57BL/6J). The number of mice analyzed was *n* = 3 for each group (young NEP^+/+^APP^−^, NEP^+/+^APP^+^ and NEP^−/−^APP^+^; aged NEP^+/+^APP^−^, NEP^+/+^APP^+^ and NEP^−/−^APP^+^). We examined brain samples from cortical areas of the frontal lobe of each mouse. 

All animal experiments were performed in compliance with the institutional guidelines for Animal Experiments of RIKEN. All procedures were conducted in accordance with the National Institutes of Health Guide for the Care and Use of Laboratory Animals. All efforts were made to minimize animal suffering and to reduce the number of animals used.

### 2.2. Light and Pre-Embedding Immunoelectron Microscopy (IEM)

The mice were perfused with a fixative containing 4% formaldehyde after being deeply anesthetized with diethyl ether inhalation. For light microscopic (LM) analysis, brain samples were immersed in the same fixative, and then embedded in paraffin. In order to be consistent with the IEM procedure and to preserve the conformation of APFs, formic acid pretreatment was not performed. For pre-embedding IEM analysis, brain tissue blocks were immersed in the same fixative, and then cut into 40 *μ*m-thick sections with a vibrating microtome. 

The brain sections for LM and EM were incubated with a conformation-dependent anti-annular protofibril (*α*APF) antiserum (1 : 500) [16], which has been well characterized by ELISA and Western blotting and selectively recognizes APFs regardless of their sequence and heptameric alpha hemolysin pores, or anti-A*β* N-terminal, affinity purified polyclonal antibody (pAb) (2 *μ*g/mL, IBL, Japan), which recognizes the amino-terminal structure of A*β* not full-length APP and beta C-terminal fragments (*β*CTFs). For LM, immunoreaction (IR) was visualized using a Vecstain ABC Elite kit (Vector Lab., USA) with a diaminobenzidine (DAB). For pre-embedding IEM, the brain sections were then incubated with horseradish peroxidase (HRP)-conjugated goat anti-rabbit IgG (Fab') (1 : 100, IBL, Japan), developed with DAB solution, osmified and embedded in epoxy resin. We tried post-embedding IEM for *α*APF antiserum, but the antiserum did not work for post-embedding IEM. On the other hand, *α*PFO pAb, A11 [1], does not work for pre-embedding IEM. Therefore, we could not compare the staining pattern between APF and PFO by the same method. For control study of LM and pre-embedding IEM, the sections were incubated with normal rabbit IgG. There was no staining of the sections incubated with normal rabbit IgG.

### 2.3. EM Observation and Quantitative Analysis

IEM sections were observed under an electron microscope (JEOL 100CXII). We randomly took 50 photographs (fields) for each mouse at the same magnification. The magnification of each photo print was × 31 000, and the actual size of each field was 2.1 *μ*m^2^. We counted the number of *α*APF-IRs and examined the distribution pattern of immunoreactions. Morphological terminology is consistent with Peters et al [20]. In addition, we counted the number of synapses in photo prints and examined whether the synaptic compartments are positive for *α*APF antiserum.

## 3. Results and Discussion

### 3.1. Light Microscopy

**Figure 1:**
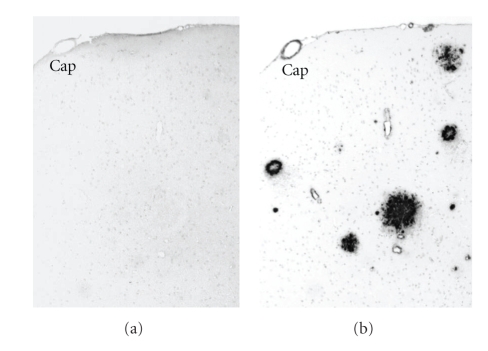
The labeling pattern of the anti-annular protofibril (*α*APF) antiserum and anti-A*β* N-terminal pAb in serial sections of aged NEP^−/−^APP^+^ mouse brain (embedded in paraffin) by light microscopy. DAB method, 100x. Cap, capillary. (a) No immunoreaction (IR) for *α*APF antiserum was observed. (b) Anti-A*β* N-terminal pAb strongly labeled senile plaques.

In all age and genotypic groups of mice, paraffin-embedded brain sections did not show any immunoreactions (IRs) for *α*APF antiserum (Figure 1(a)). This is consistent with the lack of *α*APF staining in human AD brain previously reported [16]. In comparison, anti-A*β* N-terminal, affinity purified pAb (IBL, Japan) strongly labeled senile plaques in aged NEP^−/−^APP^+^ (Figure 1(b)) and NEP^+/+^APP^+^ mouse brain sections (data not shown). Some punctate intracellular A*β* labeling was observed in aged NEP^−/−^APP^+^ and NEP^+/+^APP^+^ mouse brains (data not shown). There was no senile plaque in young mouse and aged non Tg mouse brains.

### 3.2. Ultrastructural Localization of Anti-Annular Protofibril- and A**β** N-Terminal-IRs in Mouse Brain Tissue

In young NEP^+/+^APP^−^ mouse brains, only three anti-annular protofibril-IRs (*α*APF-IRs) were observed in 150 photo prints for three mice (50 photo prints for each mouse). This means that young NEP^+/+^APP^−^ mice were almost negative for APFs at least with regard to the method used in this study. Therefore, the morphological findings mentioned below were in young Tg and aged mouse brains.

**Figure 2:**
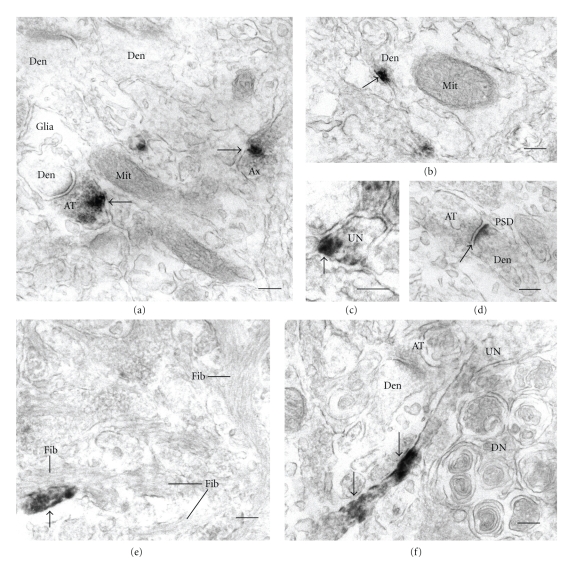
Ultrastructural localization of annular protofibrils (APFs) in aged NEP^+/+^APP^+^ (a,c) and NEP^−/−^APP^+^ (b,d–f) mouse brain sections. DAB method. (a) Anti-annular protofibril immunoreactions (*α*APF-IRs) on synaptic vesicles in axon terminal (AT) and vesicles in axon (Ax). Den: dendrite, Glia: glial process, and Mit: mitochondria. (b,c) *α*APF-IRs on plasma membranes of dendrite (b) and unidentified process (UN) (c). (d) *α*APF-IR on postsynaptic density (PSD). (e,f) *α*APF-IRs were observed on plasma membranes and vesicles inside of cell processes (arrows) in senile plaques, but not on amyloid fibrils (Fib) and distended neurites (DN). Scale bar = 200 nm.

Most of the *α*APF-IRs were localized to cell processes in a patchy pattern in young Tg and aged mouse brains at the EM level. We did not measure the area of processes and cell bodies, and did not quantitatively compare the density of *α*APF-IRs between processes and cell bodies. However, we found less apparent *α*APF-IRs inside of cell bodies during observation by EM. Axons, dendrites, and small, unidentified processes showed *α*APF-IRs (Figures 2(a), 2(b), and 2(c)). With regard to the unidentified processes, it is assumed that most were not glial but neuronal, due to their roundish appearance. Most of the *α*APF-IRs were found on plasma membranes and vesicles inside of cell processes. In synapses, *α*APF-IRs appeared on perisynaptic plasma membranes and synaptic vesicles (Figures 2(a), and 2(d)). *α*APF-positive synapses exhibited a normal appearance. Although only a few immunoreactions were observed, postsynaptic densities also showed *α*APF-IRs (Figure 2(d)). In aged NEP^−/−^APP^+^ and NEP^+/+^APP^+^ mouse brains in which many senile plaques appeared, *α*APF-IRs were not found on amyloid fibrils or in distended neurites (Figures 2(e) and 2(f)). 

**Figure 3:**
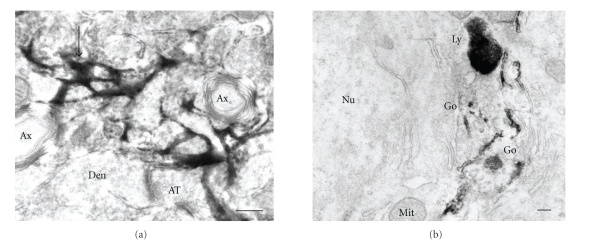
Ultrastructural localization of anti-A*β* N-terminal immunoreactions (*α*A*β* N-terminal IRs) in aged NEP^−/−^APP^+^ (a) and aged NEP^+/+^APP^+^ (b) mouse brain sections. (a) *α*A*β* N-terminal IRs (arrow, representative) were strongly observed on amyloid fibrils in senile plaques. Ax: axon, Den: dendrite, and AT: axon terminal. (b) Intracellular *α*A*β* N-terminal IRs observed on Golgi apparatus (Go) and lysosomes (Ly) in neuron. Scale bar = 200 nm. Nu: nucleus, Mit: mitochondria.

The immunoreactions of anti-A*β* N-terminal pAb were found on amyloid fibrils in aged NEP^−/−^APP^+^ (Figure 3(a)) and NEP^+/+^APP^+^ mouse brains (data not shown). Intracellularly, Golgi apparatus, vesicles in the vicinity of Golgi apparatus, lysosomes, and multivesicular bodies in neurons were positive in young and aged, NEP^−/−^APP^+^ (data not shown) and NEP^+/+^APP^+^ (Figure 3(b)) mouse brains.

### 3.3. Quantitative Analysis of **α**APF-IRs in Tg and nonTg Mouse Brains at the EM Level

**Figure 4:**
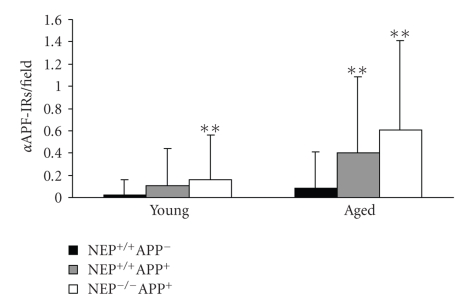
Density of *α*APF-IRs at the cell processes in mouse brain tissue. In the young group, the density was significantly higher in NEP^−/−^APP^+^ mice than that in NEP^+/+^APP^−^ mice (**, *P* < .01, Scheffe's multiple test). In the aged group, the densities were significantly higher in NEP^−/−^APP^+^ and NEP^+/+^APP^+^ mice than that in NEP^+/+^APP^−^ mice (**, *P* < .01, Scheffe's multiple test).

In the young group, the number of *α*APF-IRs per field (photo print) was significantly greater in NEP^−/−^APP^+^ (0.16 ± 0.40/field; mean ± SD) (*P* < .01, Scheffe's multiple test) than in NEP^+/+^APP^−^ mouse brains (0.02 ± 0.14/field) (Figure 4). Although the difference between NEP^+/+^APP^+^ and NEP^+/+^APP^−^ mouse brains did not reach the statistical significance, NEP^+/+^APP^+^ mice showed greater number of *α*APF-IRs per field (0.11 ± 0.33/field) than NEP^+/+^APP^−^ mice (Figure 4). In the aged group, NEP^−/−^APP^+^ (0.61 ± 0.80/field) and NEP^+/+^APP^+^ mice (0.40 ± 0.69/field) exhibited significantly greater numbers of *α*APF-IRs per field than in NEP^+/+^APP^−^ mice (0.09 ± 0.33/field) (*P* < .01, Scheffe's multiple test) (Figure 4). With regard to the age in the same genotype, the number of *α*APF-IRs per field was significantly greater in aged mice than in young mice (*P* < .05 for NEP^+/+^APP^−^ mice; *P* < .01 for NEP^−/−^APP^+^ and NEP^+/+^APP^+^ mice, Mann-Whitney's U test). 

Next, we analyzed the distribution pattern of *α*APF-IRs, which localized to cell processes (Table 1). Although the distribution pattern did not show statistically significant difference among three genotypic groups for each age (*χ* ^2^ for independence test), differences in distribution among process types were observed. In all groups of mice, many of the total *α*APF-IRs were localized to small, unidentified processes, followed by axons. The distribution of *α*APF-IRs on axon terminals was greater in aged NEP^+/+^APP^+^ and NEP^−/−^APP^+^ mice than in aged NEP^+/+^APP^−^  mice. The *α*APF-IRs were not observed on axon terminals in young mice. In addition, a few *α*APF-IRs were observed on postsynaptic process in young NEP^+/+^APP^+^ and NEP^−/−^APP^+^ mice, and aged NEP^−/−^APP^+^ mice.

Then, we counted the number of *α*APF-positive and -negative synapses in photo prints (Table 2). The aged NEP^−/−^APP^+^ and NEP^+/+^APP^+^ mice exhibited the significantly higher positive rate than that in aged NEP^+/+^APP^−^ mice (*P* < .01, *χ* ^2^ for independence test).

### 3.4. Discussion

 We have provided the first ultrastructural evidence that APFs interact with biological membranes in mouse brain tissue using pre-embedding IEM. The *α*APF-IRs increased due to aging, APP transgene and neprilysin deficiency. Furthermore, the data show that neprilysin deficiency increased the number of APFs in the synaptic compartment of Alzheimer model Tg mouse, especially in NEP^−/−^APP^+^ mouse brains.

 With regard to the IEM study, the preservation of morphology from post mortal change is particularly important. In the pre-embedding IEM, the morphology is poor compared to that in the post-embedding IEM. This is because the concentration of fixative is weaker in the pre-embedding than in the post-embedding IEM. The *α*APF antiserum works only for pre-embedding IEM, not for post-embedding IEM. Furthermore, the large post mortal change is unavoidable in autopsied human brain due to the post mortem interval. In order to obtain better morphology, we did not use human brains. In addition, we examined the influence of neprilysin on the accumulation of APFs in this study. For the purpose of this study, the brains of human APP Tg mice with or without neprilysin deficiency are better than human brains. For these reasons, we did not use human AD and control brains. 

 At the light microscopic level, paraffin-embedded brain sections did not show any immunoreactions for *α*APF antiserum. In addition, the result that *α*APF-IRs were not found on amyloid fibrils in aged NEP^−/−^APP^+^ and NEP^+/+^APP^+^ mouse brains at the EM level was remarkably different from the immunoreactions of A*β* PFOs [21, 22], which were recognized by A11 PFOs-specific antibody [1], and A*β* recognized by anti-A*β* N-terminal antibody. Although the number was few, the immunoreactions of A11-positive A*β* PFOs were found on the periphery of amyloid fibril deposits [21, 22]. These findings suggest the difference between A*β* PFOs and APFs in the pathways of A*β* aggregation. The A*β* PFOs could be not only the intermediates of A*β* fibril aggregation but also the precursors for APF formation [16]. However, APFs could not be the intermediates but seem to be on a distinct pathway from A*β* fibril formation. It is not clear that what is the determinant of the pathways for A*β* PFOs to form whether amyloid fibrils or APFs. Yamamoto et al. [23] demonstrated that cell-surface GM1-ganglioside of cultured neurons induces thioflavin-S-positive A*β* assembly. Such GM1-ganglioside-rich membrane microdomains may provide the platform for early stage of A*β* fibril aggregation. 

 Many of the total *α*APF-IRs were localized to small, unidentified processes. At the subcellular level, most of the *α*APF-IRs are localized to the plasma membranes and vesicles inside of cell processes. In previous studies, the membrane-bound A*β* was demonstrated as the initial deposition of A*β* in diffuse plaques [24]. Furthermore, A11-positive A*β* PFOs [12, 21, 22] and rafts [25] were also observed on plasma membranes in small, unidentified processes and organelles within cell processes. The similarity of APFs with PFOs and rafts in the spatial terms might account for the possibility that rafts offer the sites of initial A*β* aggregation. The interaction of PFOs with membranes catalyses their conformational conversion into annular protofibril pores [16]. Oligomerization of A*β* begins intracellularly rather than extracellularly [26, 27]. In light of these points, our findings that the APFs interact with the biological membranes, and *α*APF-IRs were observed on plasma membranes and vesicles inside of cell processes are reasonable. 

 Membrane permeabilization may represent a primary, common mechanism of pathogenesis for amyloid diseases [7, 28]. Lal et al. [29] suggested that the ion channels made of small A*β* oligomers perturb cell ionic homeostasis and exert cellular toxicity. Preformed APFs do not insert efficiently when they have been formed in the absence of the target membrane, and are significantly less toxic than PFOs. However, when PFOs interact with membranes, conducting *β*-barrel APF pores may be able to grow and elongate by integration of additional PFO subunits [16]. These results may account for PFO's ability to permeabilize membranes and APF's toxicity related to their ability to form membrane permeabilizing *β*-barrel pores [16]. 

 APP is axonally transported and cleaved by *β*- and *γ*-secretases to produce A*β* presynaptically [30, 31]. A*β* assembly is initiated at synaptophysin-positive site in primary neuronal cultures [23]. Several lines of evidence have demonstrated that the soluble A*β* oligomers are primary responsible for synaptic dysfunction in the brains of AD model Tg mice [10–12]. Neprilysin, an A*β*-degrading enzyme, localizes to presynaptic sites and contributes to clearance of A*β*, especially oligomeric A*β*, in mouse brain [18]. Neprilysin is decreased in AD brain compared to that in normal controls, and decreased neprilysin may contribute to AD pathogenesis [32, 33]. In the current study, we found significantly greater numbers of *α*APF-IRs per field, and higher positive rate of *α*APF-IRs in synaptic compartment predominate to presynaptic site in the brains of aged Alzheimer model Tg mice, especially in NEP^−/−^APP^+^ mice than in aged NEP^+/+^APP^−^ mice. In the previous study, neprilysin deficiency induced the increase of A*β* PFOs at the synapses, especially to presynaptic site, in the hippocampus and the dentate gyrus of 3-4 month-old NEP^−/−^APP^+^ mice compared to NEP^+/+^APP^+^ mice [12]. Moreover, the 3-4 month-old NEP^−/−^APP^+^ and NEP^+/+^APP^+^ mice exhibited the impaired hippocampal synaptic plasticity and cognitive function before senile plaque formation [12]. On the other hand, Lacor et al. [34, 35] demonstrated that the A*β*-derived duffusible ligands (ADDLs) bound to dnedritic spines induced aberrant spine morphology and decreased spine density using mature hippocampal neuron cultures. Long term potentiation (LTP), a form of synaptic plasticity, is closely associated with postsynaptic site involving postsynaptic phosphorylation and glutamate receptor trafficking [36, 37]. It is likely that A*β* oligomers exert their effect on postsynaptic process. The reason for the difference in the observed pre- or post-synaptic localization, in which the immunoreactions of prefibrillar aggregates of A*β* were predominantly observed, was unclear. However, due to the localization of neprilysin to the presynaptic site, neprilysin deficiency might amplify the elevation of A*β* PFOs and APFs at the presynaptic site. Our results indicate that A*β* PFOs that escape degradation by neprilysin at presynaptic site further aggregate to APFs to form pores on the plasma membranes and membranes of vesicles within cell processes. Such membrane pores might disturb ionic homeostasis and exert toxicity at cell processes and synapses. The presence of neprilysin at presynaptic site is particularly important to protect synapses from oligomer-derived synaptotoxicity. Our findings further indicate the possibility that using neprilysin activity could alleviate or improve the cognitive dysfunction in AD by reducing oligomeric A*β* at synaptic compartment.

## 4. Conclusions

In conclusion, the localization of APFs to plasma membranes and vesicles inside of cell processes indicates the possibility that these are the target and/or the production site of APFs. The increase of the positive rate of *α*APF-IRs at synaptic compartment in aged Alzheimer model Tg mice further supports the hypothesis that accumulation of A*β* oligomers including PFOs and APFs in synaptic compartments results in synaptic, cognitive dysfunction in AD. Furthermore, using neprilysin activity may have a chance to treat the cognitive dysfunction in AD by reducing oligomeric A*β* at synaptic compartment.

## Figures and Tables

**Table 1:** Distribution of anti-annular protofibril immunoreactions (*α*APF-IRs) localized to cell processes.

Process		Axon	Axon	Dendrite	Postsynaptic	Unidentified	Total^(a)^
Type			Terminal		Process		
Young	NEP^+/+^APP^−^	0(0.0%)^(b)^	0 (0.0)	1 (33.3)	0 (0.0)	2 (66.7)	3
	NEP^+/+^APP^+^	3 (18.8)	0 (0.0)	1 (6.3)	1 (6.3)	11 (68.8)	16
	NEP^−/−^APP^+^	3 (12.5)	0 (0.0)	1 (4.2)	2 (8.3)	18 (75.0)	24

Aged	NEP^+/+^APP^−^	3 (23.1)	1 (7.7)	0 (0.0)	0 (0.0)	9 (69.2)	13
	NEP^+/+^APP^+^	11 (18.3)	6 (10.0)	1 (1.7)	0 (0.0)	42 (70.0)	60
	NEP^−/−^APP^+^	16 (17.6)	12 (13.2)	2 (2.2)	5 (5.5)	56 (61.5)	91

^(a)^The number of photo prints examined was 150 for each age and genotypic group. ^(b)^The number and percentage of total*α*APF-IRs.

**Table 2:** The number and percentage of **α**APF-positive and -negative synapses.

		Positive	Negative	Total^(**a**)^
Young	NEP^+/+^APP^−^	0 (0.0%)^(b)^	255 (100.0)	255
	NEP^+/+^APP^+^	1 (0.3)	300 (99.7)	301
	NEP^−/−^APP^+^	2 (0.7)	302 (99.3)	304

Aged^(c)^	NEP^+/+^APP^−^	1 (0.4)	261 (99.6)	262
	NEP^+/+^APP^+^	6 (2.1)	283 (97.9)	289
	NEP^−/−^APP^+^	17 (5.4)	297 (94.6)	314

^(a)^The total number of synapses observed in 150 photo prints for each group. ^(b)^The number and percentage of total synapses. ^(c)^The positive rate was significantly higher in aged Alzheimer model Tg mice than in aged non-transgenic mice. *P* < .01, *χ* ^2^ for independence test.
